# Antibacterial Discovery and Development: From Gene to Product and Back

**DOI:** 10.1155/2015/591349

**Published:** 2015-08-03

**Authors:** Victor Fedorenko, Olga Genilloud, Liliya Horbal, Giorgia Letizia Marcone, Flavia Marinelli, Yossi Paitan, Eliora Z. Ron

**Affiliations:** ^1^Department of Genetics and Biotechnology, Ivan Franko National University of Lviv, Lviv 79005, Ukraine; ^2^Fundación MEDINA, Health Sciences Technology Park, 18016 Granada, Spain; ^3^Department of Biotechnology and Life Sciences, University of Insubria, 21100 Varese, Italy; ^4^The Protein Factory, Interuniversity Centre Politecnico di Milano, ICRM CNR Milano, and University of Insubria, 21100 Varese, Italy; ^5^Clinical Microbiology Laboratory, Meir Medical Center, 44281 Kfar Saba, Israel; ^6^Department of Molecular Microbiology and Biotechnology, Tel Aviv University, 6997801 Tel Aviv, Israel; ^7^Galilee Research Institute (MIGAL), 11016 Kiryat Shmona, Israel

## Abstract

Concern over the reports of antibiotic-resistant bacterial infections in hospitals and in the community has been publicized in the media, accompanied by comments on the risk that we may soon run out of antibiotics as a way to control infectious disease. Infections caused by *Enterococcus faecium, Staphylococcus aureus, Klebsiella* species, *Clostridium difficile, Acinetobacter baumannii, Pseudomonas aeruginosa, Escherichia coli*, and other *Enterobacteriaceae* species represent a major public health burden. Despite the pharmaceutical sector's lack of interest in the topic in the last decade, microbial natural products continue to represent one of the most interesting sources for discovering and developing novel antibacterials. Research in microbial natural product screening and development is currently benefiting from progress that has been made in other related fields (microbial ecology, analytical chemistry, genomics, molecular biology, and synthetic biology). In this paper, we review how novel and classical approaches can be integrated in the current processes for microbial product screening, fermentation, and strain improvement.

## 1. Introduction

Antibacterial therapy has saved millions of lives and considerably reduced the rate of premature death from bacterial infections. These achievements led to the assumption that pathogenic bacteria and the high mortality due to infectious diseases would be a thing of the past. Unfortunately, soon after the introduction of antibiotics, reports concerning the emergence of resistance started to accumulate. Antibiotic resistance mechanisms, which appear* de novo* or are transmitted among bacteria, have been well studied and described in many reviews. These include detoxification of antibiotic molecules and mutations in the designated target or, as described recently, are mediated by population-level resistance mechanisms [1]. It is now apparent that interspecies and intraspecies horizontal gene transfer of both Gram-negative and Gram-positive bacteria represent the dominant process by which bacteria become multiresistant. The selective pressure of antimicrobial use in hospitals, in communities, and in agriculture comprises the engine driving this process. Nowadays we are aware that bacterial resistance to all currently used antibiotics has emerged for both Gram-positive and Gram-negative bacteria. This threatening situation urgently calls for a concerted international effort among governments, the pharmaceutical industry, biotechnology companies, and the academic world to react and support the development of new antibacterial agents. One example of such initiative effort is the Infectious Diseases Society of America (IDSA) call to develop 10 new systemic antibacterial drugs by 2020 [2] by targeting drug development against both Gram-positive and Gram-negative bacteria. Unless serious action is taken, the acute and dangerous situation that exists today may send us back to the preantibiotic era, when there was no cure for bacterial infections. If this happens, the prophecy of Louis Pasteur will be fulfilled and “microbes will have the last word.”

## 2. Medical Needs for Novel Antibacterials

Multidrug-resistant bacterial infections represent a major public health burden, not only in terms of morbidity and mortality, but also in increased expenses for managing patients and implementing extensive infection control measures. Mortality due to multidrug-resistant bacterial infections is high. In 2002 it was reported that 1.7 million healthcare-associated infections occur each year in American hospitals and were associated with about 99,000 deaths [3]. This represents a huge increase from a previous estimation, which reported that in 1992 about 13,300 people died from hospital-acquired infection [4]. It is estimated that in the EU, Iceland, and Norway about 37,000 patients die as a direct result of a hospital-acquired infection each year; an additional 111,000 die as an indirect result of hospital-acquired infection [5]; and about 25,000 patients die from a multidrug-resistant bacterial infection.

Presently, the most frequent multidrug resistance (MDR) bacteria are* Enterococcus faecium, Staphylococcus aureus, Klebsiella pneumoniae, Acinetobacter baumannii, Pseudomonas aeruginosa*, and* Enterobacter* spp. which therefore were termed “ESKAPE” after initially being reported [6], with several reports adding* Clostridium difficile* or other* Enterobacteriaceae* [7]. Gram-positive pathogens, such as* Staphylococcus, Streptococcus, Enterococcus*, and* Clostridium*, account for a large proportion of serious infections worldwide. An increasing percentage of such Gram-positive isolates exhibit reduced susceptibility to first-line therapies [8–10], resulting in poor clinical outcomes in both community and hospital settings [10–13]; this has a significant impact on overall healthcare utilization and costs [10, 11].* Staphylococcus aureus* and* Enterococcus* spp. were found to be among the most commonly isolated pathogens in the hospital environment, and being frequently resistant to multiple drugs complicates therapy. The representative hospital “superbugs,” methicillin-resistant* S. aureus* (MRSA) and vancomycin-resistant enterococci (VRE), frequently attract mass-media attention and, in many countries, pressure is increasing to reduce MRSA and VRE infection rates. Resistance to anti-MRSA and anti-VRE drugs is uncommon; however, infections by MRSA strains resistant to glycopeptides, daptomycin, or linezolid (common anti-MRSA drugs) and by VRE strains resistant to daptomycin or linezolid (common anti-VRE drugs) are increasingly being reported, including reports of transferable resistance mechanism to these drugs among staphylococci and enterococci. In addition, reports regarding the emergence and spread of virulent clones of MRSA and* Clostridium difficile* in the community and in hospitals, respectively, have been published often. Moreover, multidrug-resistant* Streptococcus pneumonia* clones are currently considered major community pathogens in many parts of the world, although they are being challenged by new conjugate vaccines.

Although the prevalence of Gram-negative bacteria is currently somewhat lower than that of Gram-positive bacteria, it is well recognized that Gram-negative MDR infections are emerging as a threat to hospitalized patients with a significant impact on length of hospitalization, mortality, and cost [14, 15]. These include multiresistant nonfermenters, such as* Pseudomonas aeruginosa* and* Acinetobacter baumannii*, or multiresistant, extended-spectrum *β*-lactamase-producing* Enterobacteriaceae* and more recently carbapenem-resistant* Enterobacteriaceae* (CRE) of different types. Emerging resistance is due to the spread of the* Klebsiella pneumoniae* carbapenemase (KPC) and to the novel New Delhi metallo-*β*-lactamase (NDM-1). The rising crisis of multidrug-resistant Gram-negative bacteria has prompted the use of salvage therapy with colistin, an older polymyxin known to be neurotoxic and nephrotoxic [16, 17]. However, there are already reports describing isolates of several Gram-negative bacteria that are resistant to all available antibiotics, including polymyxins [18, 19].

## 3. Natural Product Discovery: The Screening Ingredients to Exploit Microbial Diversity

**Figure 1:**
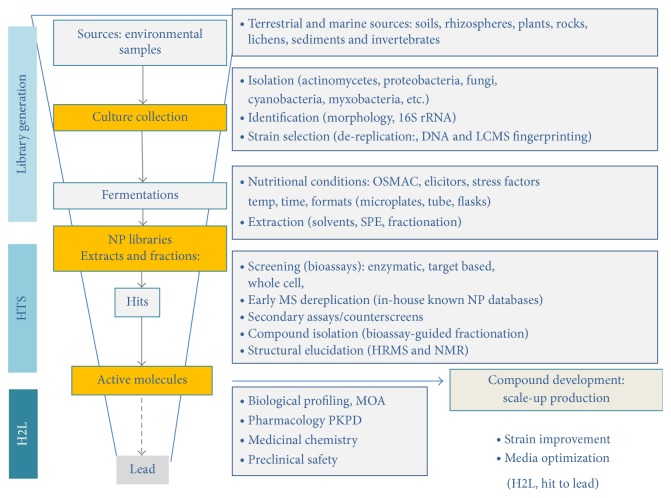
Early stages of antibiotic discovery from microbial product libraries.

Despite the pharmaceutical sector's lack of interest in addressing the topic in the last decade, microbial products continue to represent one of the most interesting sources for the discovery of novel antibacterials today and research in the field is currently benefiting from progress that has been made in other related fields (microbial ecology, metagenomics, metabolomics, or synthetic biology), fields which have provided a deeper understanding of the microbiome and thus the development of new tools to foster the discovery of novel compounds. Among living organisms, microorganisms (actinobacteria, cyanobacteria, myxobacteria, and fungi) represent one of the most prolific sources for the production of antibiotics. For decades, exploitation of their specialized (commonly termed secondary) metabolism has guaranteed the discovery of novel antibiotics and other compounds with unprecedented chemical characteristics and biological properties that do not exist in the screening libraries of synthetic compounds [20, 21]. In this section, we examine the current trends in microbial product screening for discovering novel antibiotics. A flow diagram showing the overall screening operation is reported in Figure 1.

### 3.1. Microbial Product Libraries

Microbial natural product libraries rely on the quality and diversity of novel microbial strains and the approaches used to exploit their metabolic diversity. Access to microbial diversity traditionally focused on intensive sampling and isolation using general methods from a wide range of geographical locations and habitats, with recurrent isolation and screening of the predominant species and a low probability of isolating novel compounds. Although estimates for the potential production of unknown novel molecules by* Streptomyces* spp. [22] were high, the reality is that species spread widely in different environments produce the same well-known and structurally related antibacterial molecules. Current approaches oriented to discovering novel molecules mostly aim to target specific and minor microbial communities in unique or underexplored environments, including specific terrestrial niches, plant host-microbe associations, and marine environments. Environmental conditions comprise strong selecting factors and the distribution of some microbial species, even in highly occurring taxa, presents biogeographic patterns determined by microenvironmental conditions that can be translated into novel compounds. Many research groups have recently emphasized the exploration of untapped microbial communities that are associated with rhizospheres, plant endophytes, lichens, endolithic microbial communities, insect parasites, and endosymbionts and marine sediments and invertebrates. These approaches have favored the isolation of novel microbial communities potentially producing novel chemical scaffolds [23–27]. The search for novel sources has been combined with the use of novel isolation methods targeting the cultivation of species underrepresented or previously not cultivated under laboratory conditions [28–32]. Most of these methods are focused on selective isolation of the members of minor occurring taxa by using poor nutritional media devoid of carbon sources, subinhibitory concentrations of antibiotics that might favor the development of slow-growing representatives of these microbial communities after weeks of incubation, alternative gelling substrates to agar shown to prevent the growth of some microbial groups in laboratory conditions,* in situ* incubation chambers, or isolating endophytes that germinate directly on the substrate using humid chambers or by surface sterilization.

### 3.2. Tools for Strain Selection

Strain selection criteria are essential for building a strain collection and ensure the uniqueness of the isolates and that the widest microbial diversity is represented. Phenetic and molecular tools that can be applied hierarchically on the large numbers of isolates normally recovered from environmental samples have been intensively developed. These can include a simple morphological characterization of the growth and sporulating characteristics of actinomycetes and filamentous fungi at the macroscopic and microscopic levels, allowing preliminary assignment to a taxonomic group that can be complemented with the ribosomal gene sequencing of isolates in a large proportion of the cases. Partial ribosomal rDNA sequencing is frequently used to confirm the taxonomic affiliation and to assess in molecular data the microbial diversity and individual phylogenetic relationships within strains in a collection. The existing intraspecies heterogeneity in microbial taxa cannot be resolved in phylogenetic inner branches, which require the introduction of additional fingerprinting tools for selection. Other methods currently used can include the application of high-throughput chemotaxonomic profiling methods such as those based on the whole-cell fatty acid composition [33] and the use of MALDI-TOF MS protein profiles, a promising alternative to conventional identification techniques [34], and molecular fingerprinting techniques based on the random amplification of genome-conserved repetitive regions (AFLPs, RAPDS, and REP fingerprinting) [35–37]. The generation of rapid fingerprints based on the restriction pattern of amplified conserved sequences in polyketide synthase or nonribosomal peptide synthetase biosynthetic systems provides additional information about the diversity and the biosynthetic potential of the new isolates [38].

### 3.3. Cultivation and Extraction

Traditionally the generation of microbial product libraries was based on the empiric cultivation of microbial strains in several nutritional conditions using different liquid and solid formats in varying volumes and by extracting the fermentation broths to generate crude extracts or semipurified fractions containing mixtures of specialized metabolites. The use of a limited number of three to four conditions at once, employing different media compositions, cultivation formats, or incubation periods or temperatures, was generally accepted as being sufficient to produce new, specialized metabolites, without real knowledge of the nutritional requirements and physiology of most of the groups of strains being screened and the key elements involved in regulating their specialized metabolite production. Nowadays, the continuously increasing number of whole-genome sequences of known producers shows that a large fraction of the genome remains silent and that switching on cryptic pathways might trigger the production of novel molecules [39–42]. The OSMAC (one strain, many compounds) approach has been proposed as an alternative way of exploring each strain in multiple conditions to better exploit their specialized metabolism and to trigger part of this microbial biosynthetic potential [43]. The use of multiple nutritional conditions has recently been explored by many groups to generate large screening extracts libraries in different formats (tubes, flasks), but miniaturized, parallel fermentation in deep-well plates represented a major breakthrough in the scale and numbers of conditions that can be tested [44]. All major taxonomic groups of actinomycetes and filamentous fungi can be cultivated in a large variety of complex and synthetic liquid media of diverse composition in carbon sources, inorganic or complex nitrogen sources, trace elements, and phosphate-controlled levels [20]. By testing in parallel a high number of nutritional conditions, minor groups of isolates can be explored and screened for the production of antibiotic activities. Identifying production media that can further promote their microbial biosynthetic potential increases the chances of producing novel molecules and identifying active extracts that can be then pursued on a larger scale in chemical isolation projects [45, 46].

The production of specialized bacterial metabolites can be stimulated by using known chemical inducers (e.g., siderophores, rare earths, or metabolism intermediates) [47–50], small, diffusible, bacterial, hormone-like molecules such as the *γ*-butyrolactones, and other butenolides [51]. Other elicitors of specialized metabolism include N-acetyl-glucosamine that when added to production media modulates the N-acetyl-glucosamine-responsive protein DasR [52] or generating ribosomal mutations that result in altered ppGpp biosynthesis and catabolite repression that favor biosynthesis [53]. Epigenetic modulation of fungal expression by histone acetylation and methylation has a strong influence on antibiotic production [54], and small-molecule epigenetic inhibitors of histone deacetylase (HDAC) or DNA methyltransferase (DMAT) are used to activate silent, natural product pathways in different fungal species [55, 56]. Similarly to fungi, HDAC inhibitors such as sodium butyrate or splitomicin have been reported to activate cryptic pathways in* Streptomyces coelicolor*, and HDAC orthologues have been identified to be broadly distributed in actinomycetes [57], offering new avenues to induce cryptic or poorly expressed specialized metabolites in these taxa and expand the chemical diversity of microbial product libraries.

Whereas the production conditions are key to promoting the biosynthetic potential, microbial product libraries comprise a collection of extracts and are also defined by the type of extraction used in their preparation. Extraction procedures should be designed to ensure the widest diversity of compound polarities in the extracts. These can range from simple whole-broth extraction with solvents of different polarity (from aqueous methanol or acetone miscible with the broth to more nonpolar solvents such as ethyl acetate or methyl-ethyl-ketone, providing cleaner extracts of mid-polarity compounds) to solid-phase extraction with ion exchange resins that directly enrich metabolites from the broth (cross-linked polystyrene Diaion HP20 or XAD resins) [58, 59]. Orthogonal fractionations that are used to generate prefractionated libraries reduce the complexity of the extracts, enabling screening at higher concentrations and simplifying the following dereplication phase [60].

### 3.4. Antibacterial Screening Assays

Antibiotic screening strategies of natural products have seen an important evolution in the past few decades, from the low-throughput, early phenotypic assays—used to identify compounds only targeting pathogens without any previous potential mode of action hypothesis—to high-throughput, whole-cell, target-based assays and structured-based design derived from* in silico* screening [61, 62]. High-throughput screening of microbial product libraries continues to be commonly based on phenotypic assays that have the advantage of utilizing intact bacteria and ensure that the active compound can penetrate the bacterial membranes and reach their target. Nowadays, these assays offer the possibility of integrating reporter genes to run whole-cell, target-based screens, in liquid- or agar-based format, including single- or two-plate assays, which aim to identify differential activity. The different types of assays targeting classical bacterial functions and essential pathways, including DNA replication, cell wall biosynthesis, and protein biosynthesis, have been extensively described in recent papers [63, 64]. Among these approaches, one of the breakthroughs is the use of* Staphylococcus aureus* genes essential for growth to develop a series of screens based on reducing the expression of targets to identify bacterial inhibitors. The induction of antisense RNAs to selectively decrease the production of intracellular gene products has been developed as a primary screening procedure for discovering new antibiotics [65] and was effectively employed to find novel classes of inhibitors with novel modes of action, such as the fatty acid synthesis inhibitors platensimycin and platencin, and a long list of new protein synthesis and protein secretion inhibitors [64]. An effective screening approach has consisted in the use of mechanism-based profiling using the* S. aureus* fitness test-based genome-wide screening for upfront empiric evaluation of the antimicrobial activities derived from the screening of microbial product libraries on a wide panel of bacterial pathogens [66]. The* S. aureus* fitness test consists of a collection of inducible* S. aureus* antisense RNA strains engineered for reduced expression of a single target that corresponds to essential genes for which inducible antisense RNA expression determines a growth phenotype. This assay generates a profile of strain sensitivities specific for the mechanism of action (MOA) of the compound being tested and it has been used to profile and reveal novel activities in crude microbial product extracts [63].

### 3.5. Chemical Dereplication Process

Given that known molecules continue to be rediscovered in microbial product extracts, all the HTS screening strategies have been accompanied by the implementation of efficient, early LC-MS dereplication platforms to identify known compounds in natural products databases containing known antibiotic compound spectra [67]. For identification of the bioactive compounds in microbial products extracts, bioassay-guided fractionation and further purification of the active molecule from new, large-scale refermentation of the original microbial producer are required. To miniaturize the production conditions in HTS, the desired metabolites need to be reproduced in larger fermentation formats (tubes, flasks, and bioreactors; see the following section on fermentation) later on. After confirming the original hit activity in the new extract, several rounds of chromatographic separations following the biological activity in the enriched fractions ensure that the active component has been enriched. Analysis of the active fractions by LC-MS in each round of fractionation permits dereplication of any known components that can be recognized in reference natural product databases and explains the observed activity. Normally, three to four rounds of fractionation are needed to obtain the desired molecule as a pure compound with >95% purity [68, 69]. NMR and LC-MS analytical methods are then applied not only to assess the purity of the compounds but also to generate the dossier of spectra needed to elucidate the structure of the novel compounds [60].

## 4. Fermentation Is Often the Only Way to Produce Novel Natural Microbial Products

Antibiotics are the most important category of bioactive compounds extracted from microorganisms using fermentation. During the discovery process, which is based on biologically guided screening (see the section above), sufficient amounts of active fractions need to be produced by selected microbial strains for the initial biological profiling and to elucidate the chemical structure. During the development and clinical phases, the large-scale production of antibiotics from microbial fermentations is coupled with an intensive effort to improve the strain (see the section below) in order to reduce production volumes and costs and guarantee quality and reproducibility of the drug bulks. Later, when marketing the antibiotic, which is driven by profitability and competitiveness, lower operational costs with concurrently higher yields are required for microbial production [21]. To achieve that goal, manipulating and improving microbial strains and their growing conditions (upstream process) remain the main tools since any purification scheme (downstream) at this stage is hard to improve and change due to the rigorous manufacturing regulations.

For the majority of antibiotics, the only feasible supply process continues to be fermentation, total synthesis being too complicated or too expensive.  Table 1 shows that the vast majority of the antibiotic drugs introduced into the market since 2000 are microbial products and are still produced by fermentation. Most natural products are so complex and contain so many centers of asymmetry that they probably will never be produced commercially by chemical synthesis. As an example, total chemical synthesis of the glycopeptide teicoplanin was performed by substantially inventing a new chemistry [70], but it is too expensive and microbial fermentation remains the only way to produce this valuable drug [71, 72]. However, compared to synthetic processes, manufacture by fermentation is more difficult to control; thus, it can lead to the formation of more variable antibiotic products with more complicated and less predictable composition and impurity profiles. This is due to the fact that (a) the purity of the active substances is dependent on the fungal or bacterial strains that produce the antibiotic; (b) the conditions under which strains are processed may vary; (c) the raw materials that are utilized, including the quality of water in which the strains grow, may also vary; and (d) the extraction and purification processes may have limited selectivity [73].

Hence, the crude product obtained by fermentation might not be a single antibiotic substance or entity, but rather a complex mixture of analogues, as is the case with teicoplanin (a complex of five related compounds designated teicoplanins A_2-1_–A_2-5_ characterized by five different linear or branched ten- or eleven-carbon fatty acids) [72], colistin (a multicomponent polypeptide antibiotic, comprised mainly of colistins A and B) [74], and gentamicin (oligosaccharide antibiotic composed of a mixture of three components designated as C′, C′a, and C2) [75]. Therefore, it might be difficult to compare apparently identical active ingredients unless they originate from the same manufacturer.

The need to improve the fermentation process (and reduce the cost of a multistep process) is particularly demanding for producing those natural scaffolds that undergo semisynthetic modification, as in the case of the second-generation glycopeptides (dalbavancin: trade name Dalvance, Durata Therapeutics; oritavancin: trade name Orbactiv, The Medicins Company; telavancin: trade name Vibativ, Theravance) recently approved by the Food and Drug Administration (FDA) [76].

### 4.1. Antibiotic Fermentation Process

**Figure 2:**
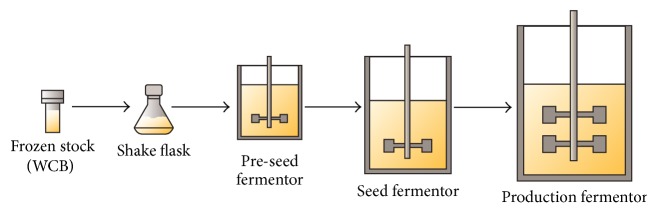
Flow diagram for the classical fermentation process: the number of seed steps may vary according to the final scale of the production fermentor.

Notwithstanding the key role of the fermentation process, not very much has changed since the first submerged fermentation process was developed to meet the demand for penicillins after the Second World War and the processes for producing antibiotics today are very similar to those employed 60 years ago. The fermentation process usually starts with a working cell bank (WCB) inoculated in a flask containing a vegetative medium (in which production does not occur) to allow the strain to grow. After a period that can vary depending on the strain, one or a series of increasing volume reactors containing vegetative medium are serially inoculated to obtain enough material to start the last-vessel fermentation within the production medium (Figure 2). Submerged fermentations for producing antibacterials are usually performed in stirred tank reactors and are operated in batch or fed-batch mode. In batch reactors all components, except gaseous substrates such as oxygen, pH-controlling substances, and antifoaming agents, are placed in the reactor at the beginning of the fermentation.

Batch processes are simple and robust, but the only way to reach a high cell density is the fed-batch mode, which is more complex but allows the metabolism of the strain to be controlled [77]. In a fed-batch process, one or more nutrients are added in order to control the reaction rate according to its concentration, avoiding catabolite repression (see below) [77]. Most antibiotics are produced with the fed-batch system (e.g., teicoplanin [72], daptomycin [78], tylosin [79], and *β*-lactams [80]) (see Table 1). Continuous culture is not common in the pharmaceutical industry because the probability of mutation and contamination is higher. Scaling up the fermentation process usually constitutes the final step in any research and development program for large-scale industrial manufacture of fermentation products [81]. Production reactor sizes range from 40 to 100 cubic meters. It is important to understand that the process of scaling up a fermentation system is frequently governed by a number of important engineering considerations and is not simply a matter of increasing culture and vessel volume.

### 4.2. Regulation of Antibiotic Synthesis and Medium Composition

Antibiotics are usually not produced during the phase of rapid growth but rather are synthesized during a subsequent stationary phase. Antibiotic production starts when growth is limited after one key nutrient source is exhausted: carbon, nitrogen, or phosphate. For example, penicillin biosynthesis by* Penicillium chrysogenum* starts when there is no longer any glucose in the culture medium and the fungus starts consuming lactose, a less readily utilized sugar [82].

The main regulation effect in specialized metabolism is, in fact, carbon catabolite repression, defined as the control (inactivation) of specific operons in favor of a primary and efficient utilization of a simple carbon source (commonly, but not always, glucose). The operons/genes/enzymes involved in crucial steps of biosynthesizing specialized metabolites are under catabolite repression. Catabolite repression is strictly linked to growth rate and growth phases since only after easily utilizable substrates have been consumed can the efficient production of specialized metabolites begin. Therefore, regulating metabolite biosynthesis ensures that precursors and metabolic energy are invested in the manufacture of specialized metabolites only under environmental circumstances and at developmental stages where those molecules contribute to the fitness of the organism [83, 84].

Glucose represses the production of many antibiotics (e.g., daptomycin [78], clavulanic acid [85], and aminoglycoside antibiotics such as streptomycin, kanamycin, neomycin, and gentamicin), but the molecular mechanism underlying glucose repression has resisted molecular analysis for a long time, although more recently this topic was thoroughly elucidated and widely covered in the literature [49, 84–87]. Readily utilizable nitrogen sources repress enzymes of specialized metabolism during the biosynthesis of cephalosporin [54, 88], cephamycin [89], tylosin [90], and erythromycin [91]. Similarly, free inorganic phosphate depletion from the growth medium is required to trigger production of tetracyclines [92, 93], *β*-lactams, and glycopeptides [93, 94]. Whereas the molecular mechanism for PhoP-mediated phosphate control is partially understood at the molecular level [93, 94], the signal sensors and signal transduction cascades involved in regulating metabolism by other stress factors need to be further elucidated [49].

To improve the production of antibiotics, slow-metabolizing carbon, nitrogen, and phosphorous sources are used: complex substrates such as polysaccharides (e.g., starch), oligosaccharides (e.g., lactose), and oils (e.g., soybean oil) are often preferred to glucose, and yeast extract, corn steep liquor, and soybean flour are commonly essential components for supplying nitrogen, phosphorous, vitamins, and trace elements to antibiotic-producing strains. In media containing a mixture of rapidly used carbon, nitrogen, and phosphorous sources and slowly used sources, the former are used first to produce cells and the latter employed once the rapidly assimilated compounds are depleted to sustain the production of specialized metabolites during the stationary phase of growth. Recent examples of how optimization of medium composition contributes to improving the final product concentration, yield, and volumetric productivity have been reported on daptomycin, nisin, cephalosporin C, clavulanic acid, and A40926, the precursor of semisynthetic dalbavancin [72, 78, 95–100]. In the case of daptomycin produced by* Streptomyces roseosporus* NRRL11379, Ng and coworkers have successfully established a cost-effective medium and feedback-controlling approach by utilizing dextrin as the major carbon source in fed-batch fermentation [78]. For glycopeptide antibiotics such as A40926 and teicoplanin, optimized media and processes have recently been proposed [65, 98–100]. The increasing list of specialized metabolism elicitors and chemical inducers, such as siderophores, rare earths, metabolism intermediates, diffusible bacterial hormone-like molecules, and N-acetyl-glucosamine, epigenetic modulators that are being used to activate cryptic or silent gene clusters during the screening processes (see previous paragraph on cultivation and extraction), can be also added to the production media to improve antibiotic production [47–57]. Limits in their use during scaling up of the fermentation process and product development consist in their cost and in the risk of chemical cross-contamination during the purification phase (downstream). Recent molecular studies have provided new insight into the role of catabolite carbon control. They demonstrated a relationship between antibiotic production and morphological development involving N-acetyl-glucosamine, which, when added to production media, modulates the N-acetyl-glucosamine-responsive protein DasR and pleiotropic regulation of both antibiotic synthesis and spore formation [52]. Molecular investigations also elucidated the role of ribosomal/RNA polymerase mutations resulting in altered ppGpp biosynthesis and in stringent response interplaying with catabolite repression [49, 101]. A thorough understanding of how global regulators (see section below on strain improvement) respond to a variety of nutritional or environmental stress signals, for example, phosphate, carbon, nitrogen starvation, heat shock, pH stress, and cell wall damage, is currently providing a more rational approach for defining medium and process conditions for antibiotic production [49, 91, 93].

## 5. Strain Improvement in the Postgenomic Era

With the development and advent of genome sequencing technologies [102, 103], it became obvious that most bacterial genomes contain a hidden wealth of clusters responsible for the biosynthesis of potential bioactive compounds [39–41] that await discovery. The main reason for the existence of such a plethora of undiscovered biosynthetic pathways is that many gene clusters are dormant or not expressed in sufficient quantities to be detected under typical fermentation conditions [104–106]. As discussed above, this is related to the existence of tight regulatory networks that precisely orchestrate specialized metabolite production in bacteria and respond to different environmental and intracellular signals [49, 86, 107]. Undoubtedly, a low yield of natural products represents a serious hurdle on the way to commercial production. Therefore, exploring and understanding the interplay between antibiotic production, regulatory networks, environmental and intracellular signals will provide us with keys to understanding specialized metabolite overproduction.

**Figure 3:**
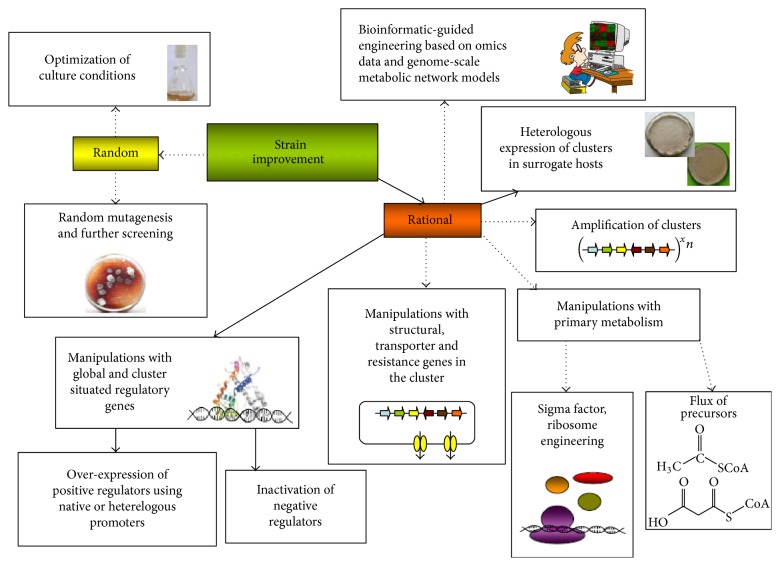
Approaches used for improving secondary metabolite production in actinobacteria. Solid arrows indicate strategies described in this review; dash-dotted arrows denote other strategies that are used.

Nowadays, numerous strategies for improving strains have been and continue to be developed. Classical approaches for strain improvement were based on recursive rounds of mutagenesis and further selection [108, 109]. Despite the drawbacks (unwanted mutations and being time consuming and laborious), this strategy was successful and widely used for rapidly increasing the production yield of antibiotic-producing microbes. Most of the industrial overproducers currently in use were developed in this way [110, 111]. However, with the development of molecular biology, biotechnology, bioinformatics, sequencing technologies, and synthetic biology, new strategies have come to the scene and provide the opportunity for rational strain improvement (Figure 3).

Overall, all of these relatively new approaches are based on spatial, temporal, and quantitative regulation of gene expression at the transcriptional or translational level, or both, thereby enabling production of higher amounts of specialized metabolites by overcoming bottlenecks, optimizing expression of genes, and redirecting the flux of precursors. Therefore, titer can be elevated by overexpressing positive regulators or deleting repressors [94, 104, 112, 113]; amplifying gene clusters [114]; redirecting the flux of primary metabolites and precursors [104–115]; overexpressing structural genes that constitute bottlenecks on the way to metabolite production [116, 117]; manipulating resistance genes and transporters responsible for the flux of antibiotic [118–120]; ribosomal engineering [101, 105]; and so forth. Substituting native promoters in a cluster with well-defined, strong promoters, either constitutive or inducible, gives an opportunity to bypass existing regulatory machinery of the host strain and improve production [121, 122]. In some cases appreciable yields of metabolites can be obtained by expressing gene clusters in surrogate hosts which are easy to manipulate (*Streptomyces lividans, Streptomyces albus*) or which are industrial strains (*Streptomyces avermitilis*) or which are genetically engineered, versatile hosts with reduced genomes (*S. avermitilis* SUKA,* Streptomyces coelicolor* M1154) [123–125]. In the following section of the review, only some examples of using regulatory genes, promoters, and heterologous hosts for rational strain improvement will be reported. Many superb and in-depth reviews have been published recently that describe different approaches for metabolic engineering of actinobacteria [104, 112, 113, 126, 127]. We refer the readers to them for a further comprehensive introduction to these topics.

### 5.1. Regulatory Genes as Basic Keys to Metabolite Overproduction

Genes involved in the production of antibiotics are located together on a chromosome or plasmid and form biosynthetic clusters. Such clusters usually contain structural, resistance, transporter, and regulatory genes. Therefore, regulatory genes that are associated with cluster and control biosynthesis of certain compound are named pathway-specific or cluster-situated regulators (CSR). They form the lowest level in the hierarchically organized regulatory network of antibiotic production in bacteria [49]. Since production of specialized metabolites is tightly connected to morphological differentiation and depends on a plethora of environmental conditions, expression of CSRs hinges on a variety of other pleiotropic, higher-level regulators that sense and transmit signals to them. In turn, CSRs, which are usually final checkpoints, transfer these signals to structural genes and switch biosynthesis of natural products on and off [49, 86]. However, like for every rule, there are exceptions in the structure of biosynthetic gene clusters. Elucidation of the genetic organization of numerous biosynthetic pathways revealed that there are some which lack CSRs [128, 129]. These findings indicate that the cluster-situated layer of regulation is not mandatory and is absent in some clusters. In such clusters, the expression of structural genes is controlled by ubiquitous regulatory genes that occupy higher levels in the regulatory web [128, 129].

According to how specialized metabolite production is influenced, all regulators can be conventionally classified into two groups: positive regulators, which activate, and negative regulators, which repress the biosynthesis of natural products. With the aim of enhancing the titer, both pleiotropic and CSRs, native and heterologous ones, are used. CSRs usually give an opportunity to manipulate one biosynthetic pathway, whereas global regulators might affect production of several specialized metabolites and/or morphological differentiation. Therefore, the effect of a pleiotropic regulatory gene very often depends on its position in the hierarchically organized regulatory network and in some cases might be unpredictable.

### 5.2. Manipulations with Positive Cluster-Situated Regulatory Genes

Overexpression of positive, pathway-specific regulators mainly enhances the transcription of structural genes responsible for the production of certain metabolites and therefore is a commonly used, single-step strategy for improving antibiotic yield. Herein, we will describe examples demonstrating the effectiveness of this approach for rational strain improvement.

*Streptomyces globisporus* 1912 is used to produce the angucycline antibiotic landomycin E (LaE). The landomycin biosynthetic gene cluster contains only one regulator gene,* lndI*, whose product is highly similar to the OmpR-PhoB subfamily of proteins. By inactivating it, antibiotic production was prevented in the I2-1 mutant, which confirms the role of LndI as an activator of LaE biosynthesis. Complementation of the I2-1 mutant with three additional copies of* lndI* gene resulted in 15-fold increase in LaE production in comparison to the wild-type strain 1912 [130], demonstrating the effectiveness of such an approach for improving the strain.

Simocyclinone D8 is an aminocoumarin compound that is produced by* Streptomyces antibioticus* Tü6040.* simReg1*, which belongs to the OmpR-PhoB subfamily of regulators, is one of three regulatory genes in the simocyclinone biosynthetic gene cluster. Its inactivation abolished antibiotic production, while overexpression of* simReg1* in an integrative pSET152-derived plasmid increased the simocyclinone titer 2.5-fold [131].

Other examples are as follows: (a) the C-1027 titer in* S. globisporus* was improved 5-fold after overexpressing the* sgcR1* gene, coding for a StrR-like protein [132]; (b) amplifying the* claR* gene encoding the LysR family protein in multicopy plasmids resulted in a threefold increase in clavulanic acid biosynthesis and in a sixfold increase in alanylclavam production [133]; (c) inserting a single copy of* pimM*, a LuxR type regulator, into the* S. natalensis* wild-type strain elevated pimaricin production 2.4-fold [134]; (d) overexpressing* fdmR1*, the encoding pathway-specific activator of the SARP family, led to a 5.6-fold increased production of fredericamycin A in* S. griseus* [135]; (e) amplifying the* tcp28* or* tcp29* genes, which encode StrR and LuxR family regulators, respectively, in the* Actinoplanes teichomyceticus* wild-type strain boosted teicoplanin production 1.5-3-fold [136, 137].

### 5.3. Manipulations with Negative Cluster-Situated Regulatory Genes

An effective and promising alternative method to overexpressing cluster-situated activators to boost antibiotic production is to inactivate pathway-specific repressors. This is exemplified by the disruption of the* lipReg3* gene coding for the MarR-type regulator that controls lipomycin export in* S. aureofaciens* Tü117, which led to a 4-fold improvement in lipomycin production in comparison to the wild-type strain [138].

Other examples that have proven the effectiveness of this strategy are as follows: (a) inactivation of the* jadR2* gene, coding a “pseudo” *γ*-butyrolactones receptor, in* S. venezuelae* generated the mutant that produces jadomycin without stress treatments (toxic concentration of ethanol, etc.) [139, 140]; (b) inactivation of another deduced *γ*-butyrolactone receptor coding gene* tylP* led to a 1.5-fold improvement in tylosin production in* S. fradiae* [141]; (c) deletion of the* ptmR1*, encoding GntR type repressor, in* S. platensis* MA7327 resulted in, on average, 100-fold overproduction of platensimycin and platencin compared to the wild-type strain [142]; and (d) inactivation of the TetR type regulator* alpW* in* S. ambofaciens* triggered constitutive production of kinamycin, a compound with antibacterial activity [143]. Thus, inactivation of repressor coding genes is useful not only for elevating antibiotic production, but, in some cases, for wakening silent gene clusters.

### 5.4. Manipulations with Pleiotropic Regulatory Genes

Successful application of omnipresent positive pleiotropic regulators to improve the titer of compounds whose biosynthetic gene clusters contain CSRs, or which are free of them, has also been shown. In most cases, a positive effect of their overexpression is due to the activation of cluster-situated regulatory gene expression or direct activation of the expression of structural genes in the cluster. For instance, overexpression of the pleiotropic regulator* afsRsv* in* S. venezuelae, S. peucetius*, and* S. lividans* TK24 led to a 4.85-, 8-, and 1.5-fold increase in pikromycin, doxorubicin, and actinorhodin production, respectively, relative to the wild type [144]. In the case of* S. venezuelae*, the increase in pikromycin production was caused by enhanced expression of the pathway-specific regulator gene* pikD* and the ketosynthase gene [144]. By introducing additional copies of the* afsR* or* afsS* genes into* S. coelicolor*, actinorhodin production could also be increased [145].

*Streptomyces ghanaensis* is a producer of phosphoglycolipid antibiotic moenomycin A [146]. The moenomycin biosynthetic gene cluster does not contain CSRs; therefore, different pleiotropic regulators were used to improve the moenomycin titer. Overexpression of the* adpAgh* gene, a pleiotropic regulator of antibiotic production and morphological development, led to a 2.5-fold improvement in moenomycin production in* S. ghanaensis* compared to the wild-type strain [129]. Introduction of the second copy of* bldAgh*, a leucyl tRNA coding gene, into the wild-type strain* S. ghanaensis* led to a 1.6-fold increase in moenomycin production [129]. Overexpression of the* relA*, a ppGpp synthetase gene from* S. coelicolor*, led to a 2-fold improvement in moenomycin production in* S. ghanaensis* relative to the wild type [147].

Similarly, inactivation of negative pleiotropic regulators in* S. ghanaensis* increased moenomycin production. The gene* absB* codes for the RNAseIII endoribonuclease involved in global regulation of morphological differentiation and antibiotic production in* S. coelicolor* [148]. By deleting it, moenomycin production was improved 2.7-fold compared with the parental strain [129]. Inactivation of another global regulator gene,* wblA(gh)*, encoding a homologue of the WhiB-family of proteins, produced a 2.3-fold increase in moenomycin biosynthesis in* S. ghanaensis* [149].

Disruption of the (p)ppGpp synthetase gene,* relA*, in* S. clavuligerus* boosted clavulanic acid production 3- to 4-fold and that of cephamycin C increased about 2.5-fold [150], confirming that there might be a pleiotropic effect of global regulator amplification or inactivation.

### 5.5. Promoters as Bio-Bricks for Titer Improvement

Another common metabolic engineering approach to induce or enhance the expression of silent or poorly expressed pathways is based on replacing native promoters in a cluster with well-defined, strong promoters, decoupling the metabolic pathway from the existing cellular regulatory networks. Examples described below clearly prove the effectiveness of the combination of two metabolic engineering strategies: amplification of positive regulators and their expression under the control of heterologous promoters of various strengths.

For this purpose different natural or synthetic constitutive or inducible promoters may be used [137, 151]. One of the most widely employed promoters in streptomycetes is the erythromycin resistance gene* ermEp* from* Saccharopolyspora erythraea* or its upregulated variant *ermEp* ^*∗*^ [152]. For example, simultaneous overexpression of the* dnrN*,* dnrI*, and* afsR* regulatory genes under the control of *ermEp* ^*∗*^ in* S. peucetius* led to a 4.3-fold increase in doxorubicin production [153]. Another prominent example of the use of this promoter is the improvement in tylosin production in* S. fradiae*. Biosynthesis of tylosin is orchestrated by the complicated interplay of five regulators [154]. To bypass existing regulatory network-positive regulators,* tylS *or* tylR* was placed under the control of the *ermEp* ^*∗*^ and overexpressed in the* S. fradiae* wild-type strain. This boosted tylosin production 3.8- and 5.0-fold, respectively [154]. Production of teicoplanin in the nonstreptomycetes actinomycete* A. teichomyceticus* was improved 2.8-fold and 10-fold by overexpressing the StrR-type regulator* tcp28* under the control of the promoter of the SARP regulator gene* actII-ORF4* and apramycin gene resistance promoter (*aac(3)IVp*), respectively, which appeared to be stronger in this strain than the widely used* ermEp* [136, 137]. This reflects the necessity to test the activity of heterologous promoters in a particular strain since their activity might differ in various species. Therefore, the repertoire of available promoters should be extended.

### 5.6. Heterologous Expression of Clusters as a Way to Overproduction

With the advent of genome sequencing and metagenomics, a plethora of clusters coding for putative biologically active compounds which previously eluded discovery because of silencing or low product yield have become and continue to become available. In addition, there are growing numbers of actinobacteria that are difficult to culture and to manipulate genetically but which produce or might produce interesting chemical compounds. The reasons outlined above drove the development of a new approach in metabolic engineering for developing surrogate high-producing hosts for the heterologous expression of gene clusters. There are a number of potential surrogate hosts. Some of them derive from well-studied* Streptomyces* strains such as* S. lividans, S. coelicolor*, or* S. albus*; others are obtained from industrial strains or are genetically engineered, versatile hosts with reduced genomes. However, the main aim of this approach is still relevant and aims to build an ideal and universal surrogate host that will be easy to genetically manipulate, is fast growing and devoid of competitive sinks of carbon and nitrogen and antibiotic activity, and will be suitable for overproduction of different specialized metabolites.

To improve moenomycin production, several* Streptomyces* strains were used as heterologous hosts, namely,* S. coelicolor* M145, M512 (Δ*actII-ORF4*, Δ*redD*),* S. lividans* TK24, 1326,* S. albus* J1074,* S. venezuelae* ATCC10712, and* S. thermospinosisporus* NRRL_B24318. The highest moenomycin titer was found in* S. albus* strains, the lowest in* S. coelicolor* [147]. These data show a high variation between different hosts. Worthy of note is that the yield of antibiotic in* S. albus* was on average 4 times higher than in the native producer* S. ghanaensis* [147].

There are several genetically engineered heterologous hosts that were obtained by controlled minimization of genomes. For example,* S. coelicolor* M1154 was constructed by deleting four gene clusters (actinorhodin, prodiginine, calcium-dependent antibiotic, and cryptic polyketide) and subsequently introducing point mutations in the* rpoB* and* rpsL* genes that enhance specialized metabolite production [125]. Expression of the gene clusters for chloramphenicol or congocidine in this strain led to a 40- and 30-fold increase in production, respectively, in comparison to the* S. coelicolor* M145 strain. Another surrogate host was developed on the basis of the industrial strain* S. avermitilis* [124]. A region of more than 1.4 Mb that contains nonessential genes and gene clusters was deleted stepwise from the chromosome of* S. avermitilis*. Expression of cephamycin C, streptomycin, and pladienolide biosynthetic gene clusters was tested in the obtained strains. Production level of streptomycin in SUKA5 strain was approximately 3 times higher than in the native producer. Biosynthesis of cephamycin C was also greatly improved. However, the biosynthesis was switched on only in the presence of the activator CcaR. Substitution of the native promoter of the* ccaR* gene with the alternative* rpsJ* promoter led to an additional increase in cephamycin C production [124], underscoring the urgency and need to use the approaches outlined above to further improve antibiotic production in genetically engineered heterologous hosts. The production of pladienolide in* S. avermitilis* engineered strains was also higher than in the* S. avermitilis* wild type [124].

Attempts to use well-studied, fast-growing, easy-to-manipulate, versatile, and widely used heterologous host such as* Escherichia coli* for the expression of actinobacterial gene clusters have also been made. This is exemplified by the production of the important antibacterial drug rifamycin. The starter unit for the RifA megasynthases is 3-amino-5-hydroxybenzoic acid (AHBA). The latter requires seven genes for biosynthesis, which are present in the rifamycin gene cluster [155]. First of all, the ability to synthesize the AHBA intermediate was reconstituted in* E. coli* BAP1. Afterwards, RifA was expressed in the AHBA-producing strain in the form of two bimodular proteins. As a result, the rifamycin intermediate P8_1-OG was synthesized at a quantity of 2.5 mg/L [155]. Other attempts to express erythromycin and oxytetracycline gene clusters in* E. coli* have also been described [156, 157]. Despite several successful tries, numerous attempts to overexpress* Streptomyces* gene clusters in* E. coli* failed. Currently, the main obstacles on the way to the desired metabolites in* E. coli* are high GC content of genes, absence of starter and extender units necessary for production, and differences in regulatory networks that generate an inability to effectively transcribe heterologous pathways. However, despite these drawbacks and taking into account a number of advantages,* E. coli* continues to be an appealing host for heterologous expression of actinobacterial gene clusters.

## 6. Conclusions

Despite the diverse classes of antibacterials that have been discovered from microbial natural product screening, there is an urgent medical need for novel molecules endowed with novel mechanisms of action to counteract emerging and multiresistant Gram-positive and Gram-negative pathogens. The microbial diversity at the origin of these novel drugs will continue to guarantee those unprecedented chemical characteristics and biological properties that did not emerge from screening libraries of synthetic compounds. Classical biological activity-based screening for novel antibacterials also relies on previous knowledge of the ecology and genome information of microbial isolates to assess their potential to produce different compounds under different cultivation conditions. Fermentation media and other parameters are being changed, taking into consideration knowledge-based use of different elicitors and tailored carbon, nitrogen, and phosphorous sources. The dramatic advances made in exploring and understanding the interplay between antibiotic production, regulatory networks, and environmental and intracellular signals are now providing us with keys to discover and overproduce new antibiotics. Currently, a wide range of genetic engineering approaches offer a large choice of tools for rational strain and fermentation improvement that might speed up the discovery and development of new, effective drugs. Combination of a growing body of knowledge in modern technologies, such as whole-genome sequencing, transcription, and metabolite profiling, offers the opportunity to make bioinformatics-based predictions of possible ways for discovering and improving specialized metabolites. Undoubtedly, further developments in functional genomics and other analytic techniques that lead to the discovery of many new signal transduction pathways and new transcription factors will reveal new, attractive targets for strain improvement approaches in the near future. In addition, approaches used in metabolic engineering continue to provide an excellent basis not only for creating overproducers, but to ensure further exploration and exploitation of the hidden part of microbial wealth. The main goal today is to develop a suite of technologies that could be used to induce the production of cryptic metabolic genes and identify previously unreported molecules, with sufficient yields to overcome one of the major problems of this century: the lack of new antibiotics.

## Figures and Tables

**Table 1:** Examples of natural products (NP), semisynthetic modified natural products (SNP), natural product-derived but produced by chemical synthesis (NP-derived), or totally synthetic antibiotics (S) launched since 2000: production method, chemical class, activity against Gram-positive and/or Gram-negative bacteria, lead source, and producing organism.

Production	Class	NP-lead source	Lead source	Antibacterial spectrum	Drug name	Year approved
Chemical synthesis	Oxazolidinone		S	G+	Linezolid	2000

Fed-batch fermentation	Lipopeptide	Actinomycete (*Streptomyces roseosporus*)	SNP (A21978C)	G+	Daptomycin	2003

Chemical synthesis	Carbapenem		NP-derived	G+/G−	Doripenem	2005

Fed-batch fermentation	Pleuromutilin	Fungus (*Pleurotus* spp.)	SNP (pleuromutilin)	G+	Retapamulin	2007

Fed-batch fermentation	Glycopeptide	Actinomycete (*Amycolatopsis* spp.)	SNP (vancomycin)	G+	Telavancin	2009

Fed-batch fermentation	*β*-lactam	Fungus (*Cephalosporium acremonium*)	SNP (cephalosporin)	G+/G−	Ceftaroline fosamil	2010

Fed-batch fermentation	Tiacumicin	Actinomycete (*Dactylosporangium aurantiacum*)	NP	G+	Fixadomicin	2011

Fed-batch fermentation	Glycopeptide	Actinomycete (*Nonomuraea* sp.)	SNP (A40926)	G+	Dalbavancin	2014
